# Acute Lung Abscess Causing Staple-Line Failure and Metalloptysis after Bullectomy: A Case Report

**DOI:** 10.5761/atcs.cr.26-00112

**Published:** 2026-07-30

**Authors:** Yuki Kamiya, Ryutaro Hanawa, Takao Shigenobu

**Affiliations:** 1 Department of Thoracic Surgery, Saiseikai Utsunomiya Hospital, Utsunomiya, Tochigi, Japan; 2 Department of Thoracic Surgery, Yokohama Municipal Citizen’s Hospital, Yokohama, Kanagawa, Japan

**Keywords:** bullectomy, lung abscess, metalloptysis, primary spontaneous pneumothorax, surgical stapler

## Abstract

Stapled bullectomy is a safe procedure for primary spontaneous pneumothorax (PSP). Early infection-driven staple-line failure with metalloptysis after bullectomy for PSP is rare. A 21-year-old man underwent video-assisted thoracoscopic bullectomy for persistent right PSP. The staple lines were reinforced with polyglycolic acid sheets. The early postoperative course was uneventful, but the patient developed fever, cough, and purulent sputum on postoperative day 21. Computed tomography demonstrated fluid-filled cavitary lesions along the staple lines in the right upper and lower lobes, with partial staple-line disruption. Sputum cultures yielded oral commensals, and an acute lung abscess associated with staple-line failure was diagnosed. Antibiotic therapy was initiated, and he expectorated several surgical staples. The abscess resolved with conservative treatment. This case suggests that acute infection along pulmonary staple lines can lead to staple-line failure and metalloptysis even after routine bullectomy. Persistent postoperative fever or purulent sputum should prompt careful evaluation for staple-line–associated infection.

## Introduction

Surgical staplers are routinely used in the treatment of primary spontaneous pneumothorax (PSP) to resect ruptured bullae or blebs. The procedure is widely regarded as safe and technically straightforward.^1–5)^ Most patients recover uneventfully and are discharged within several days. A recent nationwide registry study reported postoperative complications in approximately 12% of patients undergoing bullectomy for PSP, with prolonged air leakage being the most common complication.^6)^

Metalloptysis, defined as expectoration of metallic surgical materials, has been described as a rare complication after thoracic surgery.^7)^ Most reported cases have occurred months to years after lung resection and have been attributed to chronic foreign-body reaction, staple migration, reinforcement materials, or altered lung parenchymal architecture. However, early infection-driven staple-line failure with metalloptysis shortly after bullectomy for PSP remains exceptionally rare. Here, we report a case of acute lung abscess formation along multiple staple lines within 3 weeks after video-assisted thoracoscopic bullectomy, which resulted in staple-line disruption and metalloptysis and was successfully managed with conservative treatment.

## Case Report

A 21-year-old man with a history of infectious mononucleosis and no known allergies underwent video-assisted thoracoscopic bullectomy for persistent right PSP. Preoperative chest computed tomography (CT) showed a bulla in the right upper lobe and no apparent abnormalities in the right lower lobe (**Fig. 1**).

**Fig. 1:**
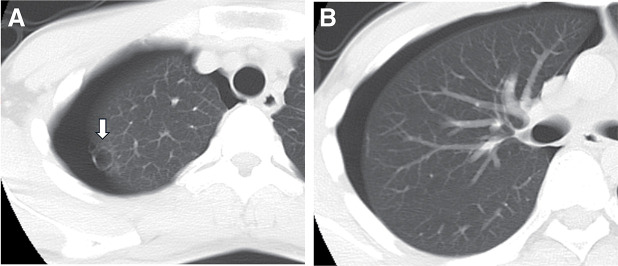
Preoperative chest CT obtained at the time of pneumothorax diagnosis. (**A**) Ruptured bulla in the right upper lobe (arrow). (**B**) Right lower lobe segment 6, where postoperative abscess formation later developed, without apparent parenchymal abnormalities. CT, computed tomography

Intraoperatively, the ruptured bulla in the right upper lobe and 2 smaller bullae in the right upper and lower lobes, which were not evident on preoperative CT, were resected using surgical staplers. The staple lines were reinforced with polyglycolic acid (PGA) sheets. Histopathology revealed no malignancy or specific inflammatory disease.

The patient’s postoperative recovery was uneventful, and he was discharged on postoperative day (POD) 2. However, on POD 8, he developed a transient fever. Chest CT revealed postoperative changes without cavitary lesions or abscess formation (**Fig. 2**). The fever resolved spontaneously within 3 days without antimicrobial therapy.

**Fig. 2:**
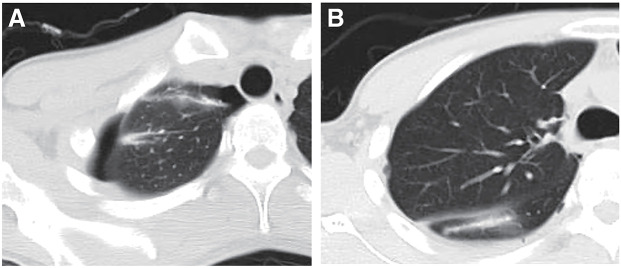
Chest CT on POD 8. (**A**) Staple lines in the right upper lobe without cavitary lesions. (**B**) Staple line in the right lower lobe without abscess formation. CT, computed tomography; POD, postoperative day

On POD 21, the patient developed fever, cough, and purulent sputum, and presented to the hospital on POD 22. Laboratory tests revealed leukocytosis (white blood cell count, 18890/μL) and elevated C-reactive protein (22.45 mg/dL). CT revealed fluid-filled cavitary lesions along the right upper lobe staple line with disruption and consolidation along the right lower lobe staple line with focal disruption (**Fig. 3**). No evidence of pneumothorax or empyema was observed.

**Fig. 3:**
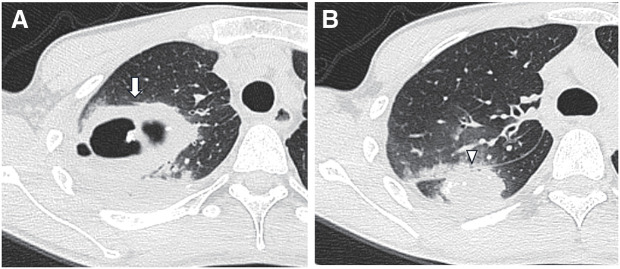
Chest CT obtained at the time of lung abscess diagnosis on POD 22, demonstrating lung abscesses associated with disruption of the staple lines. (**A**) Fluid-filled cavitary lesion along the right upper lobe staple line (arrow), with disrupted staples visible within the cavity. (**B**) Lung abscess in the right lower lobe with partial disruption of the staple line (arrowhead). CT, computed tomography; POD, postoperative day

The patient was diagnosed with acute lung abscess formation associated with staple-line disruption and was admitted for intravenous ampicillin–sulbactam therapy. Sputum cultures identified oral commensal organisms, including *Staphylococcus aureus*, *Fusobacterium nucleatum*, *Prevotella denticola*, alpha-hemolytic streptococci, and *Corynebacterium* species. Patch testing for hypersensitivity to the surgical staple, titanium, and aluminum was performed, and all results were negative.

On POD 26, the patient expectorated several surgical staples with purulent sputum. Staple expectoration continued intermittently but ceased by POD 35. Clinical symptoms gradually improved. By POD 34, inflammatory markers had normalized (white blood cell count, 5700/μL; C-reactive protein, 1.16 mg/dL). Follow-up CT demonstrated regression of the cavitary lesions. Antibiotics were discontinued on POD 36, and he was discharged on POD 37.

Sputum production gradually resolved over 6 months. Five-year follow-up CT revealed a residual cyst in the right upper lobe and complete resolution of the lower lobe lesion (**Fig. 4**).

**Fig. 4:**
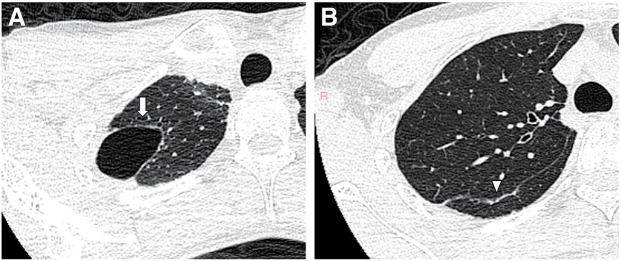
Chest CT obtained 5 years postoperatively. (**A**) Residual cystic lesion in the right upper lobe (arrow) without surrounding inflammatory changes. (**B**) Resolution of the previous right lower lobe abscess with restoration of lung parenchyma; residual disrupted staples are visible along the staple line. CT, computed tomography

## Discussion

Stapled pulmonary resection is a fundamental technique in thoracic surgery. In a multicenter retrospective study from the Central Japan Lung Cancer Surgery Study Group, 10908 lung parenchymal staplings were analyzed, and adverse events occurred in 0.74% of staplings.^8,9)^ Reported complications included prolonged air leak and bleeding; lung abscess formation or staple expectoration was not observed. Therefore, acute infection-related failure of pulmonary staple lines appears to be an uncommon event.

The clinical course in the present case suggests that the transient fever on POD 8 and the subsequent infectious event on POD 22 represented different processes. The POD 8 fever resolved spontaneously, and CT showed no cavitary lesions or abscess formation. This episode may have reflected a nonspecific postoperative inflammatory response or a localized reaction to the PGA sheet used for staple-line reinforcement. Absorbable mesh reinforcement has been reported to induce foreign-body reactions characterized by inflammatory cell infiltration and multinucleated giant cell formation.^10)^ In contrast, the POD 22 episode was accompanied by high fever, a marked inflammatory response, purulent sputum, polymicrobial culture results, and CT findings of cavitary lesions along multiple staple lines, supporting a diagnosis of acute lung abscess formation.

Shai et al. reported a clinically similar case after wedge resection for organizing pneumonia in a 40-year-old man with a history of hepatitis C.^11)^ Cough and hemoptysis developed 1 week after surgery, and pneumonia-like consolidation with air bronchograms around the surgical staple-line area was identified 1 month postoperatively. The symptoms persisted for 11 months, and bronchoscopy eventually revealed metallic staples that had migrated and become impacted at the entrance of the left upper lobe bronchus. Although that case was also considered possibly related to a foreign-body reaction to staples, it differed from the present case in the presence of underlying organizing pneumonia, the prolonged clinical course, and the need for bronchoscopic removal. Another relatively early complication after stapled bullectomy was reported by Horio et al.^12)^ They described intrapulmonary hematoma formation around the staple line 3 months after video-assisted thoracoscopic bullectomy for spontaneous pneumothorax. Although that case is relevant as an early postoperative lesion occurring around a staple line after bullectomy, the lesion was hemorrhagic rather than infectious in nature and was not associated with metalloptysis. In contrast, the present case developed acutely within 3 weeks after bullectomy for PSP and involved acute abscess formation along multiple staple lines, overt staple-line disruption, and spontaneous expectoration of staples.

In addition to these early or relatively early complications, late-onset abscess formation around staples has also been reported. Watanabe et al. described a lung abscess that developed around a staple line 11 months after partial lung resection in a patient with a permanent tracheostoma.^13)^ Chronic airway contamination related to the tracheostoma was considered a possible predisposing factor, and the lesion mimicked lung cancer recurrence. Although that case is relevant as a previous report of staple-line–associated lung abscess, the delayed onset and the presence of a permanent tracheostoma differ substantially from the present case.

Titanium hypersensitivity and foreign-body reactions have been implicated in staple-related inflammatory pulmonary lesions. Matos et al. reported inflammatory pseudotumor formation with titanium staple expectoration after pneumonectomy; patch testing demonstrated hypersensitivity to titanium salts, and the patient improved with systemic corticosteroid therapy.^14)^ In the present case, patch testing for the surgical staples, titanium, and aluminum was negative, making systemic metal hypersensitivity less likely. Nevertheless, a localized foreign-body reaction not detectable by patch testing cannot be completely excluded. The involvement of multiple staple lines and improvement after cessation of staple expectoration suggest that a local reaction to the staples or reinforcement material may have contributed to tissue vulnerability, followed by secondary infection with oral commensal organisms. Other reports have described late-onset foreign-body granulomas or pseudotumor-like lesions around staple or suture lines several months after pulmonary resection.^15–17)^ These entities differ from the present case in both timing and mechanism.

Metalloptysis has been reported as a rare and delayed complication after pulmonary stapling procedures, particularly after lung volume reduction surgery or apical bullectomy with reinforcement material.^7,18–20)^ These cases generally occurred months to years postoperatively and were attributed to chronic foreign-body reaction, staple migration or bronchial erosion, bovine pericardial reinforcement, or altered lung parenchymal architecture rather than acute infection. In contrast, early postoperative metalloptysis, as observed in the present case, is exceedingly rare. Its occurrence within 3 weeks after bullectomy for PSP, together with an abscess formation and overt staple-line disruption, suggests an acute infection-driven mechanism distinct from delayed metalloptysis reported previously.

## Conclusions

Acute lung abscess formation along pulmonary staple lines can cause staple-line disruption and metalloptysis even after routine bullectomy for PSP. Although metalloptysis has typically been described as a delayed complication related to chronic foreign-body reaction or altered lung architecture, the present case suggests that acute infection-driven staple-line failure may represent a distinct mechanism. Thoracic surgeons should recognize that similar complications may occur not only after pneumothorax surgery but also after other pulmonary resections using surgical staplers. Persistent postoperative fever or purulent sputum after pulmonary stapling should prompt careful radiologic evaluation of the staple lines.

## Declarations

### Use of Generative AI and AI-assisted technologies in the writing process

During the preparation of this work, the authors used ChatGPT to improve language clarity and readability. The authors take full responsibility for the content.

### Data availability

The data underlying this case report are not publicly available to protect patient privacy. Further information may be available from the corresponding author upon reasonable request and with appropriate institutional approval.
